# In rabbits with cheyletiellosis is topical selamectin or injectable ivermectin a more effective treatment?

**DOI:** 10.18849/ve.v7i4.529

**Published:** 2022-12-20

**Authors:** Juliette Mouëzy+

**Keywords:** CHEYLETIELLA INFECTION, CHEYLETIELLA PARASITIVORAX, CHEYLETIELLOSIS, INJECTABLE IVERMECTIN, PET RABBIT, RABBIT MEDICINE, TOPICAL SELAMECTIN

## Abstract

**PICO question:**

In rabbits with cheyletiellosis is topical selamectin or injectable ivermectin a more effective treatment?

**Clinical bottom line:**

**Category of research:**

Treatment.

**Number and type of study designs reviewed:**

One paper directly answered the PICO question, a retrospective clinical design study, and was critically reviewed.

**Strength of evidence.:**

Strong.

**Outcomes reported:**

Both topical selamectin and injectable ivermectin are safe and effective in treating cheyletiellosis. There is no significant difference in the effectiveness of both drugs in treating the infestation.

**Conclusion:**

In pet rabbits with *Cheyletiella parasitivorax* infestations both topical selamectin and injectable ivermectin have been recognised to be a safe and effective treatment. There is currently insufficient evidence that one is more effective than the other and therefore veterinarians should consider their own clinical experience, client preference and use the licensed product first (in the UK, ivermectin (Xeno, Dechra) spot-on). However, this Knowledge Summary does not comment on the effectiveness of topical ivermectin in treating cheyletiellosis. If injectable ivermectin is not available, topical selamectin can be used under the cascade as an alternative treatment, as it is licensed for the same indication in dogs and cats.

## How to apply this evidence in practice

The application of evidence into practice should take into account multiple factors, not limited to: individual clinical expertise, patient’s circumstances and owners’ values, country, location or clinic where you work, the individual case in front of you, the availability of therapies and resources.

Knowledge Summaries are a resource to help reinforce or inform decision making. They do not override the responsibility or judgement of the practitioner to do what is best for the animal in their care.

## The evidence

One paper was reviewed in this Knowledge Summary, a retrospective clinical design study (Mellgren & Bergvall, 2008). Two other papers were also found to be relevant to this Knowledge Summary as, although they do not address the PICO question directly, they evidence the individual effectiveness of topical selamectin and injectable ivermectin in treating cheyletiellosis in rabbits (Kim et al., 2008; and Coşkunserçe et al., 2012). They are discussed in the appraisal section. These studies found both injectable invermectin and spot-on selamectin to be effective in treating the infestation.  

Weaknesses in the evidence were based around the lack of more comparative studies between the effectiveness of topical selamectin and injectable ivermectin in treating cheyletiellosis and a short follow-up period. Overall, the strength of the evidence found was strong.

## Summary of the evidence

**Mellgren & Bergvall (2008):** 

Population:	Across two practices (Gästrike Animal Clinic, Sandviken, Sweden and Animal Clinic Roslagstull, Stockholm, Sweden), medical records from 282 rabbits were reviewed. Of these, 53 rabbits fulfilling prerequisites for selection were included in the study.
Sample size:	53 rabbits.
Intervention details:	Prerequisites for selection were: clinical signs compatible with *Cheyletiella *infestation; diagnosis confirmation of *Cheyletiella *mites through light microscopy examination of skin scrapings, material from flea combing or transparent tape preparations under 4 or 10 × 100 magnification; rabbits were treated with ivermectin or selamectin; a follow-up had to be possible with the owner via telephone or through a revisit at the clinic. The following information was collected from the two veterinary settings: age; sex; weight; clinical signs; diagnosis verification; treatment protocol (substance, dose, route of administration and interval); number of rabbits in the household; other treatments; evidence of concurrent diseases. The rabbits were divided into three treatment groups. For all groups, where rabbits were from multi-rabbit households, all in-contact rabbits were treated.Group 1: Ivermectin injections (Ivomec®vet. injectable, 10 mg/ml, Merial SAS, Lyon). 11 rabbits (females n = 3; males n = 8), all treated at the Gästrike Animal Clinic, with a mean age of 4.4 years (range 9 months to 7 years) and bodyweights ranging from 1.4–4.6 kg. 9/11 were from single-rabbit households. The rabbits were treated with ivermectin injections subcutaneously (SC) at two (n = 5) or three (n = 6) occasions. The mean dose was 0.253 mg/kg(range 0.200–0.476 mg/kg) and the mean injection interval was 11 days (range 9–21 days). Group 2: combination of injections and oral administration of ivermectin (Ivomec®vet. injectable, 10 mg/ml, Merial SAS, Lyon). 27 rabbits (females n = 4; intact males n = 20; castrated males n = 3), all treated at the Animal Clinic Roslagstull, with mean age of 4 years (range 6 months to 9.5 years) and bodyweights from 1.6–6.5 kg. 24/27 lived in single rabbit households, and the rest in households with two or more rabbits. Treatment consisted of three to six ivermectin administrations at 10 day intervals: the initial subcutaneous (mean dose of 1.044 mg/kg, range 0.618–2.185 mg/kg) were followed by two oral administrations of ivermectin by the owner, using the injectable formula (mean dose of 1.324 mg/kg, range 0.616–2.732 mg/kg). 23/27 rabbits were re-examined 30 days (range 28–35 days, one after 48 days) after the first visit. Based on clinical signs, 2/27 were given no additional treatments, 21/27 had a second injection, (14/27 of which had two additional oral ivermectin treatments repeated, with the same doses and original intervals) after being considered to still have clinical signs (8/27) or to be mite positive after microscopic examination (4/27). Group 3: topical selamectin (Stronghold®/Revolution, 60 mg/ml, Pfizer Inc., New York). 15 rabbits (females n = 5; intact males n = 8; castrated males n = 2), one treated at the Gästrike Animal Clinic and 14 at the Animal Clinic Roslagstull; with a mean age of 2.2 years (range 3 months to 7 years) and bodyweights ranging from 1–7.4 kg. 2/15 rabbits belonged to multi-rabbit households. Treatment consisted of administration of selamectin spot on topically at one to three occasions. The mean dose of selamectin used was 12.5 mg/kg (range 6.2–20.0 mg/kg). Treatment results were graded as in remission (free from clinical signs at re-examinations during the whole follow-up period), relapse (being free from clinical signs more than 3.5 months after treatment but showing signs again during the follow-up time) or treatment failure (cases that never cleared from clinical signs during the first 3.5 months or were recorded with relapse during this time) at the time of follow-up. Adverse reactions of treatment were assessed by clinical examination during revisit and by questioning the owner. Statistical analysis was made by using χ2 test.
Study design:	Retrospective clinical design study.
Outcome Studied:	Determine the effectiveness of three different protocols using selamectin (Stronghold®/Revolution, 60 mg/ml, Pfizer Inc., New York) and ivermectin (Ivomec®vet. injectable, 10 mg/ml, Merial SAS, Lyon) in treating cheyletiellosis in rabbits.
Main Findings(relevant to PICO question):	Rabbits in remission were 9/11 (81,8%), 14/27 (51.9%) and 12/15 (80%) in groups 1, 2 and 3, respectively. One rabbit from group 1 did experience pain on injection. From group 1, one was classed as treatment failure and one was recorded as relapse. From group 2, five rabbits were graded as treatment failures, still showing clinical signs after treatment with three to six ivermectin doses, and seven were classed as relapses. From group 3, only one rabbit was recorded as a treatment failure and two relapsed. When comparing all treatment groups with each other no significant differences were found (p = 0.09, n = 53). Rabbits with concurrent disease or that were overweight were seen in both treatment failure and remission groups, making concurrent health conditions not the main determinant of treatment effectiveness. Results of this retrospective study suggest that both injectable ivermectin and topical selamectin are effective and safe for clearance of clinical signs of cheyletiellosis in rabbits. In the group including an oral administration of ivermectin (group 2) the treatment was shown to be less effective in comparison to groups 1 (ivermectin injections) and 3 (selamectin spot-on), although not statistically significant.
Limitations:	Bias of uncontrolled events and factors such as treated rabbits being asymptomatic carriers of *Cheyletiella* mites as treatment response was in some cases assessed just by clinical cure and not parasitical cure. Owner bias and the effect that poor compliance (difficulty to medicate the rabbit at home) or drug storage (e.g. ultraviolet (UV) protection) has on the cure rate of group 2. The environment not being a controlled factor of the study also affects sources of contamination / re-contamination. Short follow-up time, not allowing for further visits to further validate the results of this study. Decision to categorise a rabbit as ‘in remission’ after only a phone call or a clinical examination and not systematic parasitology. Lack of information regarding the mean treatment time-span for rabbits enrolled in group 3.

## Appraisal, application and reflection

Although only one article was found to directly answer the PICO question (Mellgren & Bergvall, 2008), two other articles were also found to be relevant to this Knowledge Summary (Kim et al., 2008; and Coşkunserçe et al., 2012), as they allowed the comparison to be drawn regarding the effectiveness of either selamectin or ivermectin in infested rabbits. Whilst the heterogeneous nature of the study designs (one being a prospective case report and the other a clinical design study), rabbit populations and recorded data precludes direct comparison of their results with each other; these articles present clear evidence for the benefit of using either topical selamectin and injectable ivermectin to treat cheyletiellosis in rabbits.

Mellgren & Bergvall’s (2008) retrospective case study, in addition to being the most relevant article available to treat our PICO question, brings strong evidence in support of the efficacy of both drugs in treating cheyletiellosis in rabbits by comparing the effect of three different treatment programmes on a sample base from across two practices. The effectiveness of both topical selamectin (Stronghold^®^/Revolution, 60 mg/ml, Pfizer Inc., New York) and injectable ivermectin (Ivomec^®^vet. injectable, 10 mg/ml, Merial SAS, Lyon) was confirmed, whilst doubts about the success rate of oral ivermectin in treating cheyletiellosis were raised. Despite variable treatment success rates between groups, this was not found to be statistically significant. The short length of follow-up time, and variables of the study (environment, client bias) were its biggest limitations. The variability of thoroughness of the post-treatment assessment also raised some risks of re-infection through shedding of mites by ‘in remission’ rabbits, re-infecting themselves, causing a relapse. A replication of this study with a larger cohort, more control over external parameters, a longer follow-up period with clinical examinations and strict microscopic examinations, would allow for a more thorough interpretation of the comparative effectiveness of both drugs.

The prospective clinical design study led by Kim et al., (2008) evaluated the effectiveness of a single application of spot-on selamectin (Revolution®, Pfizer; Kalamazoo, MI, USA) in treating cheyletiellosis across a cohort of 23 pet rabbits. Signs of improvement were evaluated at 3 and 5 weeks post-treatment through clinical and microscopic examination of epidermal debris for mites and eggs. Complete cure was obtained after 5 weeks post-treatment for the whole group and no relapse was noted 12 weeks post-treatment, suggesting the effectiveness of a single spot-on application to treat the infestation. Although successful, the lack of statistical analysis and control over external factors were the main limitations of the study. However, if the study was to be repeated with a large cohort size and addressed some of its limitations it would be a great advocate for the use via the cascade of spot-on selamectin. With no side effects resulting from its application it would be a more client and pet friendly (as no injection is required) alternative to injectable ivermectin when treating cheyletiellosis.

Finally, in the case study led by Coşkunserçe et al. (2012), a single rabbit was treated for *C.parasitivorax* with subcuntaneous (SC) ivermectin (Iverkol, Etkin) injection to assess the effectiveness of high dose (1.2 mg/kg ) treatment in treating cheyletiellosis. The rabbit was then examined every 10 days for clinical sign improvements and every 7 days for skin scrapes to assess the presence of live mites. After day 14, no clinical signs nor microscopic signs of infestation were noted. However, as positive as the outcome of this case report is, and it describes the effectiveness of high doses of ivermectin in treating cheyletiellosis, it has many limitations: only one animal enrolled, descriptive approach but no statistical analysis of the relevance of the results, and the bias of interpretation as no grading system was used to assess the improvement of clinical signs. This study is relevant, as it demonstrates the effectiveness of ivermectin (Iverkol, Etkin) injections in the treatment of cheyletiellosis, however, it would need to address certain limitations if it was to be replicated to give more weight to its findings.

Despite numerous limitations within each study, evidence supports that both injectable ivermectin and topical selamectin are efficacious in treating cheyletiellosis in rabbits. Effectiveness of avermectins in controlling rabbit mange has been reported in previous studies for moxidectin, selamectin, doramectin, and ivermectin (Niaz & Shoaib, 2015). Altogether the effectiveness of the treatment, low rate of re-infestation and improvement of clinical signs, indicate that the use of both drugs can be warranted in treating cheyletiellosis. Both drugs have their individual benefits, topical selamectin being client friendly and non-painful as it is delivered as a spot on (Farmaki et al., 2009) and injectable ivermectin having proven efficacy and relying less on client compliance (Mellgren & Bergvall, 2008). Currently, injectable ivermectin is not licensed for use in rabbits and therefore, unless prescriptible under the cascade, topical selamectin should be used for the treatment of cheyletiellosis in rabbits (Robinson & Brennan, 2016). Although it has been seven years since their publication, there is no further evidence on this topic. However, with the risk of development of resistance, ivermectin should be used judiciously in treatment of rabbit mange (Coşkunserçe et al., 2012).

Additional well-designed (prospective, controlled, randomised) studies comparing the effectiveness of topical selamectin and injectable ivermectin in larger groups are needed to further evaluate the benefit of both drugs. Further research into the pharmacodynamics and bioavailability of each drug in rabbits post-treatment would also inform comparative results, assuming the literature is available, in helping a clinician choose between topical selamectin or injectable ivermectin (under the cascade).

## Methodology

**Search Strategy:** 

Databases searched and dates covered:	CAB Abstracts on OVID Platform 1973Surrey Open Search, University of Surrey, 1990–2020 PubMed accessed via the NCBI website (1990-2020, filtered for Veterinary Science) Science Direct, Elsevier, 1990–2020
Search strategy:	CAB Abstracts: “Rabbit” and “treatment” and “Cheyletiellosis” “Rabbit” and “treatment” and “mange mites” (“comparative treatment” or “treatment efficacy”) and (“ivermectin” and “selamectin” or “avermectins”) and (‘’Cheyletiella parasitivorax’’ or ‘’cheyletiellosis’’ or ‘’cheyletiella infection’’ or ‘’mites’’) and “rabbit” “comparative treatment” and (“ivermectin” and “selamectin”) and “mange mites” and “rabbit” Surrey Open Search: “Rabbit” and “treatment” and “Cheyletiellosis” “Rabbit” and “treatment” and “mange mites” “comparative treatment” and (“ivermectin” and “selamectin”) and “cheyletiellosis” and “rabbit” “comparative treatment” and (“ivermectin” and “selamectin”) and “mange mites” and “rabbit” PubMed: “Rabbit” and “treatment” and “Cheyletiellosis” “Rabbit” and “treatment” and “mange mites” “comparative treatment” and (“ivermectin” and “selamectin”) and “cheyletiellosis” and “rabbit” “comparative treatment” and (“ivermectin” and “selamectin”) and “mange mites” and “rabbit” Science Direct: “Rabbit” and “treatment” and “Cheyletiellosis” “Rabbit” and “treatment” and “mange mites” “comparative treatment” and (“ivermectin” and “selamectin”) and “cheyletiellosis” and “rabbit” “comparative treatment” and (“ivermectin” and “selamectin”) and “mange mites” and “rabbit”
Dates searches performed:	03 May 2022

**Exclusion / Inclusion Criteria:** 

Exclusion:	Papers that do not answer the PICO question. Papers published prior to 1990. Papers studying the use of ivermectin or selamectin in animals other than rabbits. Papers that do not focus on treatment of cheyletiellosis but on identification; parasitology and other factors of the disease. Papers that discuss the use of other drugs in the treatment of cheyletiellosis.
Inclusion:	Relevant papers, including papers comparing the effectiveness of selamectin and ivermectin in the treatment of cheyletiellosis in rabbits, with reproducible methods, a detailed approach and measurable results.

**Search Outcome:** 

Database	Number of results	Excluded – that do not answer the PICO question	Excluded – study compared the use of other drugs	Excluded – study did not compare selamectin / ivermectin in rabbits	Excluded – study not discussing the treatment of cheyletiellosis	Total relevant papers
CAB Abstracts	39	15	8	14	1	1
Surrey Open Search	129	45	22	50	11	1
PubMed	123	112	5	0	5	1
Science Direct	1759	482	415	416	445	1
Total relevant papers when duplicates removed						1

## ORCID

Juliette Mouëzy: https://orcid.org/0000-0002-5838-9384

## Conflict of interest

The author declares no conflicts of interest.
